# Level of colorectal cancer awareness: a cross sectional exploratory study among multi-ethnic rural population in Malaysia

**DOI:** 10.1186/1471-2407-13-376

**Published:** 2013-08-07

**Authors:** Tin Tin Su, Jun Yan Goh, Jackson Tan, Abdul Rahim Muhaimah, Yoganathan Pigeneswaren, Nasirin Sallamun Khairun, Abdul Wahab Normazidah, Devi Kunasekaran Tharisini, Hazreen Abd Majid

**Affiliations:** 1 Centre for Population Health (CePH), Department of Social and Preventive Medicine, Faculty of Medicine, University of Malaya, Kuala Lumpur 50603, Malaysia; 2 Faculty of Medicine, University of Malaya, Kuala Lumpur, Malaysia

**Keywords:** Colorectal cancer, Rural population, Cancer awareness, Bowel/colorectal CAM

## Abstract

**Background:**

This paper presents the level of colorectal cancer awareness among multi-ethnic rural population in Malaysia.

**Methods:**

A rural-based cross sectional survey was carried out in Perak state in Peninsular Malaysia in March 2011. The survey recruited a population-representative sample using multistage sampling. Altogether 2379 participants were included in this study. Validated bowel/colorectal cancer awareness measure questionnaire was used to assess the level of colorectal cancer awareness among study population. Analysis of variance (ANOVA) was done to identify socio-demographic variance of knowledge score on warning signs and risk factors of colorectal cancer.

**Results:**

Among respondents, 38% and 32% had zero knowledge score for warning signs and risk factors respectively. Mean knowledge score for warning signs and risk factors were 2.89 (SD 2.96) and 3.49 (SD 3.17) respectively. There was a significant positive correlation between the knowledge score of warning signs and level of confidence in detecting a warning sign. Socio-demographic characteristics and having cancer in family and friends play important role in level of awareness.

**Conclusions:**

Level of awareness on colorectal cancer warning signs and risk factors in the rural population of Malaysia is very low. Therefore, it warrants an extensive health education campaign on colorectal cancer awareness as it is one of the commonest cancer in Malaysia. Health education campaign is urgently needed because respondents would seek medical attention sooner if they are aware of this problem.

## Background

The colorectal cancer is the third most common cancer worldwide. It has been one of the most common cancers in developed countries and becoming more apparent in developing countries [1]. In Malaysia, colorectal cancer (CRC) is the most common cancer among males and third most common cancer among females [2,3]. The CRC is a largely preventable disease which requires community participation in the prevention process, such as life style modification and regular medical screening [4].

Most cancers arise from as a result of a complex interaction between genetic and environmental factors. The risk factors for CRC also includes increasing age, positive family history, low dietary fibre, high saturated fat intake, red meat consumption, excess alcohol, lack of physical activity and having diabetes mellitus [5-11].

The 5-year survival rate of CRC can be as high as 90% if the disease is detected early [12]. It is therefore important to screen for those who are at risk of colorectal cancer which may help in minimising its’ mortality rate. An earlier study in Malaysia which was conducted in a teaching hospital, has shown that the awareness of colorectal cancer screening is almost nil among the in-patients who were newly diagnosed with colorectal cancer [13]. Due to poor awareness, the possibility of late detection was high. Even though if the patients with rectal bleeding (which one of the warning signs for CRC), a study has shown these patients still delaying in seeking advice from the medical personnel [14]. It is crucial that community should be aware of the risk factors and warning signs and symptoms on CRC. This may lead them to be actively involved in the screening process due to adequate knowledge about the disease with positive perception about it [9].

Despite realizing increasing trend of CRC, health promotion regarding this disease is not highlighted by the Ministry of Health compared to other cancers such as lung, cervical and breast cancer. Furthermore, no national screening programme was adopted for the colorectal cancer. It is, therefore, important to measure the colorectal cancer awareness in the Malaysian population as a basis for considering possible changes in practice. With the urbanisation, Malaysia had experienced a large increase in its urban population and aging society but about 37% of population are still living in rural area [15]. According to the nationwide survey conducted in 2006, it has shown that awareness and screening practices for cancer were low in rural area and in some states, where it was reported that the rural community faced difficulty in accessing to the health care facilities [16].

In view of the apparent clinical benefit of screening in the prevention of CRC, it is important to establish the community understanding about CRC. Hence, this survey was conducted to investigate the level of CRC awareness among the rural population in Malaysia.

## Methods

### Study setting

A rural-based cross sectional survey was carried out in Perak state in Peninsular Malaysia in March 2011. Perak encompasses of nine districts where mainly agriculture and some commodity based manufacturing are the state economic drive. This study was approved by the Ethics committee, University Malaya Medical Centre (Ref. no. 890.6). The sample size was determined by using the OpenEpi programme. Estimated rural population in Perak was one million and based on the assumptions that 50% (+ or - 5) of rural population has sufficient knowledge of bowel cancer with 95% confidence interval and 80% power of the study, the calculated sample size was 385 individual. Since the bowel/colorectal cancer awareness questionnaire was administered as one of modules in the household survey, we decided to collect the data from all adults who were selected for household survey in order to increase the precision of the study.

**Figure 1:**
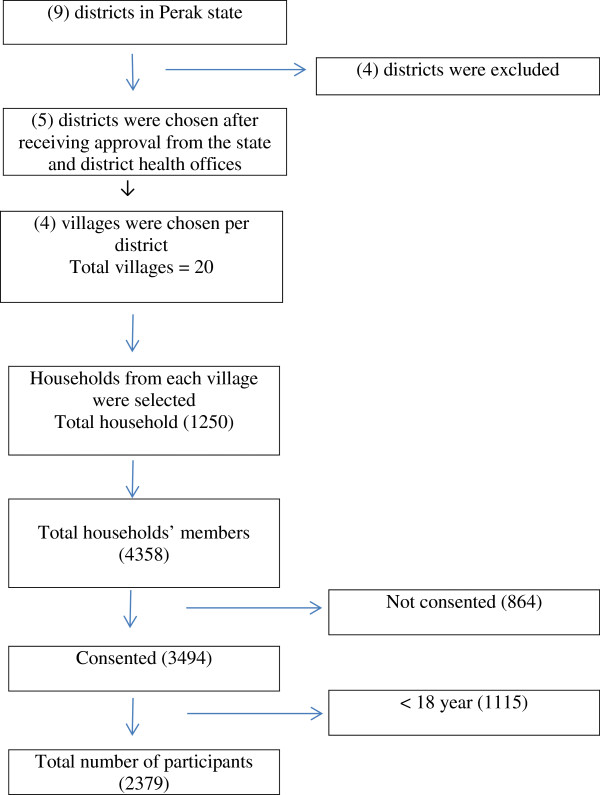
Study flow chart for bowel cancer awareness measurement.

The survey recruited a population-representative sample using multistage sampling. The five districts of the Perak state (Kampar, Kuala Kangsar, Taiping, Parit Buntar and Gerik) were selected. At the second stage, four villages were chosen per district. Both districts and villages were chosen purposively with discussion with state and district health offices. Finally, households were selected from villages with simple proportionate random sampling. From the village maps, the sampling frames were constructed by tagging every household with a serial number. Selection of households was done by using a computer generated random number table. A total number of 1250 households were included in the study (Figure 1).

### Study tool

The level of colorectal cancer awareness was accessed by using the Bowel/Colorectal Cancer Awareness Measure (Bowel/Colorectal CAM) questionnaire. The survey instrument was developed by the Health Behaviour Research Centre, UK. It is based on a generic CAM developed by Cancer Research UK, University College London, King’s College London and Oxford University in 2007-2008 [17,18]. There are 25 specific questions on bowel cancer awareness in original Bowel/Colorectal CAM. Among 25 items, only one question concerning NHS bowel cancer screening programme was excluded since it is not suitable for Malaysian context. The rest of the questions were included in the survey. The survey instrument (Bowel/Colorectal CAM) was translated to Malay, national language. Forward and backward translation was performed by independent individuals.

Face validity was done with 30 participants before conducting survey. The participants evaluated: 1) whether the questionnaire measures what it intends to measure in terms of the comprehensiveness and clarity of the questionnaire; 2) whether the questionnaire is simple, easily understood, any inappropriate, redundant or missing items, and how likely the questionnaire was to address the research objective; 3) the relevancy, flow and arrangement of the questionnaire; and 4) the wording of the questionnaire.

#### Knowledge of warning signs and symptoms of colorectal cancer

There are 1 unprompted item (open-ended question) and 9 prompted (close-ended questions) on warning signs and symptoms of colorectal cancer. The “open-ended” question is designed to measure how many colorectal cancer warning signs a respondent can recall unaided. The knowledge scale of warning sign was assessed by the “close-ended” questions. The stem question for the knowledge scale of warning signs is phrased as; “The following may or may not be warning signs for bowel cancer. We are interested in your opinion”. This is followed by the list of nine warning signs (bleeding from back passage, pain in abdomen, change in bowel habit, feeling of incomplete emptiness of bowel, blood in stool, pain in back passage, lump in abdomen, tiredness/anaemia and unexplained weight loss) each of which can be identified as a warning sign for bowel cancer or not. A scoring system for the warning signs was used where each appropriate answer (Yes) is given a point according to the previous study conducted in UK [19].

#### Knowledge of risk factors of colorectal cancer

There are 1 open-ended question and 10 close-ended questions on risk factors of bowel cancer. The “open-ended” question is designed to measure how many colorectal cancer risk factors a respondent can recall unaided. The knowledge scale of risk factors was assessed by the “close-ended” questions. The stem question for the knowledge scale of risk factors is phrased as; “The following may or may not increase the chance of developing bowel cancer. How much do you agree that each of these can increase the chance of developing bowel cancer?” This is followed by the list of ten risk factors (‘Drinking more than 1 unit of alcohol a day’, ‘Eating less than 5 portions of fruit and vegetables a day’, ‘Eating red or processed meat once a day or more’, ‘Having a diet low in fibre’, ‘Being overweight or obese’, ‘Being over 70 years old’, ‘Having a close relative with bowel cancer’, ‘Doing less than 30 minutes of moderate physical activity 5 times a week’, ‘Having a bowel disease’, ‘Having diabetes’ with response options of “strongly disagree, disagree, not sure, agree and strongly agree”. The similar scoring system was used as above where each appropriate answer (agree or strongly agree) is given a point.

The remaining questions covered self-rated confidence level of noticing bowel cancer (4 Likert score: “not at all confident: 1” to “very confident: 4”), health seeking behaviour and opinion on age related bowel cancer incidence. The socio-demographic information and having of any cancer for self, partner, family and friend were asked.

#### Description of variables

The total knowledge score for warning sign of bowel cancer ranges from 0 to 9 and the total knowledge score for risk factor ranges from 0 to 10. Other variables were socio-demographic characteristics that included age, gender, ethnicity, marital status (single, married, divorce and widow), education level (primary/secondary and post-secondary) and monthly income. Having incidence of cancer in self/partner/close family members; other family members; among friends were also used as explanatory variables.

#### Process of data collection

Fifty trained interviewers (25 pairs) did the face –to- face structured questionnaire interview. All interviewers were third year University students and fluent in both English and Malay. The training of the interviewers was performed in five sessions by the principle investigator. The context of the study, proper way of conducting survey and standardized method of data entry were explained and discussed during training sessions. The first and last authors checked the results carefully in order to control the interviewers’ bias. Participation in the study was voluntary and the written informed consent for participation in the study was obtained from participants.

### Statistical analysis

Collected data was entered and analysed using the Stata version 11 (StataCorp LP, TX, US). The data is then checked for outliers, errors and omissions and cleaned. Descriptive and bivariate analyses were done as preliminary data analysis. Association between knowledge score of colorectal cancer and independent variables was analysed by using ANOVA. Analysis was performed for awareness of symptoms and risk factors separately.

## Results

In the 1250 households, there was a total of 4358 household members and out of that, 3494 consented to involve in the survey, making the response rate 80.2%. Colorectal cancer awareness was only assessed in respondents aged 18 and above at the day of interview. Out of the 3494 respondents, 2379 adults who fulfilled the age criteria were recruited in the study. The description of the study participants are presented in (Table 1).

**Table 1:** Socio-demographic characteristics of the respondents (N = 2379)

**Socio-demographic characteristics**		**Mean (±Std Dev) or percentage (%)**
Age in years		51.1(±16.9)
Gender	Male	43.3
	Female	56.7
Ethnicity	Malay	78.3
	Chinese	14.0
	Indian/Others	7.7
Marital Status	Single/Never Married	14.2
	Married	76.3
	Divorced/Separated	0.9
	Widowed	8.6
Highest Education Level	Primary/Secondary	93.4
	Post secondary	6.6
Monthly Income	Below RM 1000	44.1
	RM 1000 and above	55.9

### Knowledge of warning signs and symptoms

Among 2379 participants, 74% could not recall any warning sign without aided. Average recall was less than one warning sign and symptom (mean 0.44; SD 0.92). “Abdominal pain” was the most commonly recognized warning sign for CRC where 14.5% of the respondents could recall. It was followed by “bleeding from back passage” and “blood in stool”. About 4% of respondent managed to recall “change in bowel habit” “feeling of incomplete emptiness of bowel” and “tiredness/anaemia”. Very few participants could answer “unexplained weight loss”, “lump in abdomen”, and “back passage pain” as possible warning signs of bowel cancer. The awareness of warning sign and symptoms measured by unprompted and prompted questions are summarized and presented in (Table 2). The prompted awareness for all warning sign and symptoms was higher than unprompted.

**Table 2:** Awareness of warning signs and symptoms (N = 2379)

**Signs and symptoms**	**Unprompted (%)**	**Prompted (%)**
Abdominal pain	14.5%	36.1%
Bleeding from back passage	6.6%	37%
Blood in stool	6%	40.6%
Change in bowel habit	3.9%	28.4%
Feeling of incomplete emptiness of bowel	4.1%	26.7%
Tiredness/Anaemia	4%	27.7%
Unexplained weight loss	2.3%	33.1%
Lump in abdomen	2%	35.5%
Back passage pain	1.4%	24.6%

### Knowledge of risk factor

Similarly, unprompted awareness of risk factors for CRC were very poor, with average recall of less than one risk factor (mean 0.48; SD 1.18). Approximately 77% percent of respondents could not recall any risk factors without aided. “Low intake of fruits/vegetables” was the most commonly recognized risk factor for bowel cancer where 11.6% of the respondents could recall. It was followed by “low fibre diet”, “high intake of red and processed meat” and “alcohol consumption”. “Low physical activity” was identified as a risk factor by 3.4% of respondents. About 3% of respondent managed to recall “family history of having bowel cancer” “old age” and “being overweight or obese”. Very few participants could answer “having other bowel disease”, and “having diabetes” as possible risk factors of bowel cancer. The awareness of risk factors measured by unprompted and prompted questions are summarized and presented in (Table 3). The prompted awareness for all risk factors was higher than unprompted.

**Table 3:** Awareness of risk factors (N = 2379)

**Risk factors**	**Unprompted (%)**	**Prompted (%)**
Low intake of fruit/vegetables	11.6%	39.4%
Low fibre diet	8.8%	36.2%
High intake of red and processed meat	7.7%	35.1%
Alcohol consumption	5.6%	45.6%
Low physical activity	3.4%	32.7%
Family history of having bowel cancer	3%	31.2%
Older age	2.9%	33.6%
Being overweight or obese	2.8%	32.5%
Having other bowel disease	1.8%	41.2%
Having diabetes	1.1%	21.8%

### Knowledge of age related incidence of colorectal cancer

The respondents were asked that in the next year, who is most likely to develop colorectal cancer. The choices of answers are “a 20 year old, a 40 year old, a 60 year old and bowel cancer is unrelated to age”. Only 17% of the participants could give the right answer that a 60 year old is likely to develop bowel cancer. Fifty six percent responded that bowel cancer is unrelated to age.

### Knowledge score of colorectal cancer

The knowledge score of CRC was derived from the close-ended questions for warning signs and symptoms, and risk factors. Among respondents, 38% and 32% had zero knowledge score for warning signs and risk factors respectively. Mean knowledge score for warning signs and risk factors were 2.89 (SD 2.96) and 3.49 (SD 3.17) respectively. Twenty seven percent of the respondents had zero knowledge score for CRC and mean total knowledge score was 6.38 (SD 5.45).

### Confidence in noticing a warning sign and Help-seeking

A majority of the respondents (over 60%) were not confident in their own ability of noticing a warning sign of CRC. Almost thirty percent were fairly confident and only 5.3% were very confident in noticing warning signs. There was a significant positive correlation (Pearson correlation constant = 0.201, p < 0.01) between the knowledge score of warning signs and level confidence in detecting a warning sign.

Among the respondents, 87.6% would seek help within one week if presented with warning signs of bowel cancer and the mean duration for seeking help after noticing possible sign of bowel cancer was 1.51 (±1.69) weeks. Ethnic difference in anticipated delay in help seeking was found. Among study participants, 10% of Malay, 7% of Chinese and 20% of Indian and others had anticipated delay for help seeking (Pearson chi^2^ = 24.6303, P =0.000).

### Factors associated knowledge score on warning signs and risk factors

Socio-demographic variance of knowledge score on warning signs and risk factors were identified by ANOVA and presented in (Table 4).

**Table 4:** Socio-demographic variance of knowledge score on warning signs and risk factors (N = 2379)

	**Warning signs**		**Risk factors**	
	**Mean (95% confidence interval)**	**Analysis of variance (ANOVA)**	**Mean (95% confidence interval)**	**Analysis of variance (ANOVA)**
**Age**				
18–19 (n = 75)	2.77 (2.08 – 3.46)	F (30.13)	3.00 (2.27 – 3.72)	F (19.11)
20–29 (n = 279)	3.51 (3.14 – 3.88)	P < 0.001	4.08 (3.71 – 4.45)	P < 0.001
30–39 (n = 275)	3.82 (3.47 – 4.17)		4.18 (3.82 – 4.53)	
40–49 (n = 431)	3.52 (3.14 – 3.80)		4.09 (3.80 – 4.39)	
50–59 (n = 539)	3.00 (2.75 – 3.25)		3.59 (3.32 – 3.86)	
>60 (n = 780)	1.93 (1.74 – 2.11)		2.67 (2.45 – 2.89)	
**Gender**				
Male (n = 1030)	2.82 (2.65 – 3.00)	F (0.99)	3.53 (3.33 – 3.72)	F (0.31)
Female (n = 1349)	2.94 (2.78 – 3.11)	P > 0.05	3.46 (3.29 – 3.63)	P > 0.05
**Ethnicity**				
Malay (n = 1863)	3.09 (2.96- 3.23)	F (21.77)	3.70 (3.55 – 3.84)	F (19.70)
Chinese (n = 332)	2.32 (2.02 – 2.62)	P < 0.001	2.62 (2.31 – 2.94)	P < 0.001
Indian/Others (n = 184)	1.87 (1.47 – 2.27)		2.91 (2.43 – 3.38)	
**Marital status**				
Single (n = 330)	3.19 (2.85 – 3.52)	F (8.2)	3.60 (3.25 – 3.95)	F (6.7)
Married (n = 1815)	2.95 (2.82 – 3.09)	P < 0.001	3.59 (3.44 – 3.75)	P < 0.001
Divorce/separated (n = 21)	1.80 (0.49 – 3.12)		1.85 (0.73 – 2.97)	
Widowed (n = 204)	2.02 (1.63 – 2.42)		2.71 (2.29 – 3.12)	
**Education**				
Primary & secondary (n = 2222)	2.77 (2.65 – 2.90)	F (55.43)	3.38 (3.25 – 3.51)	F (38.04)
Post-Secondary (n = 157)	4.54 (4.08 – 5.00)	P < 0.001	4.99 (4.51 – 5.47)	P < 0.001
**Income**				
< RM 1000 (n = 1783)	2.62 (2.49 – 2.76)	F (58.53)	3.24 (3.09 – 3.38)	F (45.34)
≥ RM 1000 (n = 596)	3.68 (3.45 – 3.92)	P < 0.001	4.24 (3.99 – 4.49)	P < 0.001

In the age group of 60 and above, there was a lowest knowledge score for both warning sign and risk factors. Compared to Malay, Chinese and Indian participants had significantly lower knowledge of CRC. Similarly, compared to single and married, divorce/separated and widowed had significantly lower knowledge score for both warning signs and risk factors. Gender was not significantly associated with awareness of CRC.

Respondents who had post-secondary education had higher awareness of CRC. Having monthly income RM 1000 and above had significant association for having better knowledge score compared to low income group.

Having cancer in self/spouse/close family had significantly higher knowledge of warning sign but not for risk factors of CRC. Having cancer in other family member and friends had significant association for having better knowledge score for both warning sign and risk factors (Table 5).

**Table 5:** Association of previous experience of cancer and knowledge score on warning signs and risk factors (N = 2379)

	**Warning signs**		**Risk factors**	
	**Mean (95% confidence interval)**	**Analysis of variance (ANOVA)**	**Mean (95% confidence interval)**	**Analysis of variance (ANOVA)**
**Having cancer in self/spouse/close family**				
Yes (n = 2076)	3.71 (3.37 – 4.06)	F (27.16)	3.38 (3.24 – 3.52)	F (18.36)
No (n = 303)	2.77 (2.64 – 2.90)	<0.001	4.22 (3.87 – 4.56)	< 0.001
**Having cancer in other family member**				
Yes (n = 2201)	3.62 (3.19 – 4.05)	F (11.86)	4.19 (3.76 – 4.61)	F (9.31)
No (n = 178)	2.83 (2.71 – 2.95)	< 0.001	3.43 (3.30 – 3.50)	< 0.01
**Having cancer in friends**				
Yes (n = 2024)	4.05 (3.73 – 4.36)	F (65.16)	4.63 (4.32 – 4.94)	F (54.96)
No (n = 355)	2.69 (2.56 – 2.81)	<0.001	3.29 (3.15 – 3.43)	< 0.001

## Discussion

Understanding and recognising public awareness regarding CRC may provide valuable information to incorporate the policy decision for prevention, early diagnosis and improvement of survival for CRC. At the moment, data regarding colorectal cancer awareness has not been described in Malaysian population especially in the rural context.

Results from the current study demonstrated that a large number of population have poor knowledge regarding sign and symptoms of colorectal cancer. More than 70% of the interviewed subjects could not recall any sign unaided. Clinical presentation of colorectal cancer varies and often non-specific [20]. An earlier hospital based study discovered that anaemia and weight loss were two common clinical symptoms presented among CRC patients in Kuala Lumpur [21]. However, these non-specific colorectal cancer symptoms are difficult to be differentiated from other diseases by these population especially who resided in rural areas. Among our study population only less than 5% could relate these symptoms to colorectal cancer without aided. Even for prompted question, less than one third of the population agreed that anaemia and weight loss as warning signs and symptoms of CRC. This would delay seeking medical professionals help.

Rashid et.al (2009) also found out that abdominal pain is the third common clinical presenting symptoms in CRC patients in one of the teaching hospital in Malaysia [21]. This was consistent with current findings. The abdominal pain was the most recalled signs unaided and similarly, it was second most agreed sign of CRC from prompted question. It appears the rural community perceived abdominal pain was related to CRC due to its’ anatomical site compared to anaemia and weight loss. Back passage pain was the least well recalled signs and symptoms for CRC. Despite having the pain which was perceived as haemorrhoids, earlier studies have shown even though patients experiencing rectal bleeding, due to poor symptoms recognition, they delayed in seeking medical treatment [22,23].

From this study, the mean duration for seeking medical attention was 1.51 (±1.69) weeks. This is within the acceptable range as seeking treatment more than two weeks after notice of warning sign was considered as delay [24]. Current finding showed majority of the population would actually seek medical attention within two weeks provided if there are aware with the sign and symptoms of CRC. This provides an opportunity for the Ministry of Health Malaysia to do more campaign to create awareness among general population so that it would prevent delay seeking treatment.

The rural population in Malaysia had minimal awareness regarding poor lifestyle behaviour as risk factors for CRC. These include low fruit and vegetables intake, low fibre, high intake of red meat and processed meat, being overweight and low physical activity. These basics key messages for health promotion had been highlighted in World Cancer Research Fund Report (2007) and these are the key issues that should be addressed in public health context. A study in China has shown men are at greater risk of CRC than women possibly due to their unhealthy lifestyle habits and poor fruits and vegetable consumption [25]. However, in the current study, gender was not significantly associated with awareness of CRC. In addition, poor awareness about the role of physical activity in preventing CRC in current study also similar to the finding in the US [26]. It is important that the healthcare professionals should take this opportunity to play an active role communicating the messages for cancer prevention through lifestyle modification at the health care facilities [27] and through the mass media. In Malaysia, rural community have the access to the television and this should be used for education tool. A study in Malaysia has shown one of the best methods in educating the rural population was via mass media for example television [28] where ninety seven per cents of the population received information regarding severe acute respiratory syndrome during the outbreak via television.

Respondents with higher education level and income in this study, have a higher level of awareness on CRC. This is consistent with findings from previous studies in the United Kingdom which reported respondents from affluent groups had shown higher level of cancer awareness [17,29]. Additionally, those who had experienced cancer themselves and those with friends who have cancer showed a higher level of knowledge of CRC. There is a possibility that they are more familiar with the disease because they may have heard about it from their family or friends hence raising their awareness. However, it would be good if every community members are aware of CRC even if they live in rural areas as this is possible to do [30].

According to the National Cancer Registry, incidence of bowel cancer was highest among the Chinese where the incidence rates was 23.8 per 100000 populations, and lower in Indians and Malays where the incidence rate was 9.1 per 100000 and 6.9 per 100000 respectively [3]. Due to this, it can be assumed that the Chinese should have better awareness on CRC as it is more common in their population. However, from this study, the Chinese have significantly lower level of awareness when compared to Malays. Several possible reason contributed to such findings which includes the information from the national registry are based on patients who seek treatment which may be under represented by other ethnic group who did not seek for medical treatment. Majority of available information about bowel cancer in public health care facilities are in English and Malay. Some other ethnic group may not understand the health information as they could not speak both languages.

There are several strengths with our study. This is the first study using validated Cancer Awareness Measurement (CAM) questionnaire conducted in Asia which included the participants came from multi-ethnic rural population. Findings from our study can show the level of CRC awareness among rural population in a middle income country. By using a standardised questionnaire, international comparison of CRC awareness will be possible.

The data for this study were collected from the rural population because of our particular interest on health promotion among rural population. However, findings of our study limits to rural population and cannot be generalized to the whole Malaysian population.

## Conclusions

The level of awareness on CRC warning signs and risk factors in the rural population of Malaysia is very low. It is crucial to conduct health promotion progamme to increase awareness as to encourage public to seek for medical attention if they have these symptoms. This warrants multi-component intervention from all stakeholders for prevention of CRC in the rural population.

## Competing interests

The authors declare that they have no competing interests.

## Authors’ contributions

The first and last authors designed the study and did translating and validation of the study instrument. All authors contributed in data collection, analysis and write-up of manuscript. All authors read and approved the final manuscript.

## Pre-publication history

The pre-publication history for this paper can be accessed here:

http://www.biomedcentral.com/1471-2407/13/376/prepub
